# Synthesis, *In Vitro* Antioxidant Activity, and Physicochemical Stability of Antioxidant-Containing Nanospheres and Their Effects on Biodiesel Oxidation Stability

**DOI:** 10.1021/acsomega.6c00993

**Published:** 2026-05-19

**Authors:** Eduarda Carolina Hagemann Lopes, Karen Cristine Silva de Oliveira, Giovano Tochetto, Fernanda Oliveira Lima, Dalila Moter Benvegnú, Letiére Cabreira Soares, André Lazarin Gallina

**Affiliations:** † Federal University of Fronteira Sul (UFFS), Campus Realeza, Postal Office Box 253, Avenida Edmundo Gaievski, 1000, Rodovia BR 182—Km 466, 85770-000 Realeza, Paraná (PR), Brazil; ‡ Department of Chemistry, Midwestern Paraná State University (UNICENTRO), Alameda Élio Antonio Dalla Vecchia, 838, Center for Technological Development of Guarapuava, 85040-167 Guarapuava, Paraná (PR), Brazil

## Abstract

Biodiesel is highly susceptible to oxidation due to its chemical structure, which necessitates antioxidants. This study developed a controlled-release system using poly-ε-caprolactone (PCL) nanospheres containing *tert*-butyl-hydroquinone (TBHQ) and ascorbic acid (AA) to provide long-term protection against environmental degradation. Consequently, the physicochemical stability and antioxidant activity of these systems were monitored over 150 days. The results demonstrated the successful production of stable nanospheres (polydispersity index < 0.3) that effectively shielded the compounds. TBHQ-loaded nanospheres retained over 80% antioxidant activity, while AA-loaded versions maintained more than 50% activity during the 150-day study at 30 °C. Furthermore, the antioxidant-loaded nanospheres were tested in biodiesel samples. Unexpectedly, adding these nanospheres reduced the induction time compared to pure biofuel. This reduction may be attributed to a hypothesis that nanometric surface imperfections on the spheres serve as active sites which potentially promote radical oxidation reactions. However, further surface analyses are necessary to confirm this hypothesis. Ultimately, these results suggest that PCL nanospheres excel as long-term storage carriers, and that their morphological interaction with the biodiesel matrix requires further investigation to fully understand the observed effects on induction time.

## Graphical abstract

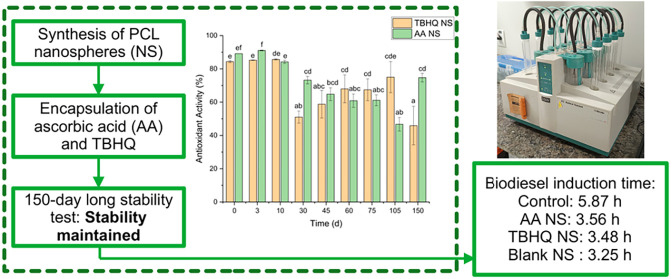

## 1 Introduction

Currently, nonrenewable sources such as coal, natural gas, and petroleum meet most of the world’s energy demand.^1^ Using these energy sources causes several negative environmental and health impacts, most notably the large-scale emission of greenhouse gases.^2–4^ Derived from petroleum, diesel is widely used in diesel cycle engines, which are typically found in heavy vehicles and electric generators. Biodiesel, which is usually produced from the transesterification of triglycerides in vegetable oils, is a viable substitute for diesel, offering the primary advantages of renewability and lower pollutant emissions.^5,6^

In this context, the environmental benefits of biodiesel justify global efforts to promote its production. However, its chemical properties can cause undesirable degradation, including oxidation. This susceptibility stems primarily from unsaturated fatty acids, which oxidize when exposed to heat, light, metals, moisture, or oxygen. This exposure initiates radical reactions, forming peroxides that propagate the process. These intermediates eventually form polymers that can clog fuel systems, increase fuel viscosity, and generate acids, both of which accelerate engine wear.^7–9^

Moreover, antioxidants minimize or inhibit biodiesel oxidation by neutralizing free radicals,^10,11^ reducing oxygen concentrations, and sequestering metal ions.^12^ These compounds are classified as either synthetic or natural. Synthetic antioxidants, such as *tert*-butylhydroquinone (TBHQ), are widely preferred for their high efficiency in maintaining oxidative stability.^12^ Conversely, recent studies have highlighted the potential of natural antioxidants, including tocopherols, phenols, and flavonoids, found in fruit peels and leaf extracts.^13–17^ Additionally, ascorbic acid (AA) inhibits free radicals through effective electron donation.^18^ Beyond stabilization, antioxidants can provide secondary benefits, such as increased lubricity and reduced emissions.^19,20^

Antioxidants added to biodiesel may degrade at high temperatures, reducing their effectiveness.^21^ Encapsulating these substances is a promising strategy to overcome this challenge.^22–24^ In this context, polymeric nanoparticles are widely used for controlled delivery in diverse fields, particularly in controlled drug delivery.^25^ Specifically, nanocapsules feature a polymeric membrane surrounding an oily core containing the active substance. In contrast, nanospheres (NS) consist of a polymeric matrix that retains the active agent without an internal oily core.^26,27^

Nanoparticles may offer several advantages for use in biodiesel, primarily by protecting the active substances from environmental stress and enabling their controlled release. This significantly enhances antioxidant efficiency, as demonstrated in related fields.^22,28^ If such effects are also observed in the prevention of oxidation of biodiesel, the application of antioxidant encapsulated nanospheres would represent a valuable strategy. While studies evaluating free antioxidant solutions and natural extracts are abundant,^29–35^ developing nanocarriers is essential to ensure prolonged protection and optimize the delivery of these active agents.

Despite their potential, antioxidant-loaded polymeric nanoparticles in biodiesel remains an under-explored frontier, with existing literature being notably sparse. Current literature focuses predominantly on engine performance and emission profiles, where nanoparticles generally yield beneficial outcomes.^36–41^ However, the physicochemical stability of these additives and their influence on oxidative kinetics have been largely neglected. Addressing this gap, the present study developed and characterized poly-ε-caprolactone (PCL) NS containing AA and TBHQ. Moving beyond the current state of the art, this research evaluates the NS long-term antioxidant activity over an extended 150-day period, and investigates their effects on biodiesel oxidation stability.

## 2 Results and Discussion

Table 1 presents the physicochemical results for the blank, TBHQ and AA NS along with their respective encapsulation efficiency (EE).

**Table 1:** Zeta Potential, PDI, Size, and EE for Blank, TBHQ and AA NS (*n* = 3)

Sample	Zeta Potential (mV)	PDI	Size (nm)	pH	EE (%)
Blank	–10.25 ± 0.65	0.189 ± 0.032	277.07 ± 2.90	4.76	-
TBHQ	–12.50 ± 0.42	0.158 ± 0.028	267.25 ± 4.03	6.20/7.7	64.33
AA	–11.63 ± 0.21	0.245 ± 0.035	401.20 ± 9.37	3.26/3.57	18.80

Nanoparticle size distribution homogeneity was assessed by the polydispersity index (PDI), which ranges from 0 to 1. A PDI value near zero signifies a highly homogeneous system, whereas a value closer to one indicates a heterogeneous distribution.^14,26^ As shown in Table 1, the TBHQ NS exhibited the highest degree of homogeneity, followed by the blank NS. Conversely, the AA-loaded NS displayed the lowest homogeneity. A possible explanation is that the acid groups of unencapsulated AA may interact electrostatically with the NS surfaces, leading to colloidal instability, larger particle sizes, and higher PDI values.^42^ Similar findings were reported by Jang and Lee,^43^ who observed that both the PDI and size of chitosan nanoparticles increased with higher AA concentrations.

Furthermore, the nanoparticle sizes are appropriate for this application, as they align with typical values for polymeric systems, and biodiesel stabilization does not require a specific size range. Despite the absence of strict size requirements, the AA-loaded NS diameters are consistent with similar reports. For instance, the largest particles in this study are comparable to those synthesized by Liu et al.^44^ who used a double emulsion method with dichloromethane to produce poly­(lactic acid-*co*-glycolic acid) (PLGA) NS containing daunorubicin. Similarly, Mahmoud and McConville^45^ reported nanospheres of approximately 200 nm using the same methodology, which closely matches the results obtained in this work.

As an EE of 50–80% is acceptable according to the literature,^46,47^ the observed EE is considered acceptable for this method, considering the TBHQ encapsulation. Conversely, the observed EE for AA (18.80%) is notably lower. From a formulation perspective, a low EE implies that a significant fraction of the AA remains as “free” solute, which may induce an initial burst release effect rather than a strictly controlled-release profile. Furthermore, regarding scalability, a low EE requires a higher initial mass of AA to achieve the desired loading, which could increase production costs. Despite these limitations, this value is sufficient to demonstrate a proof-of-concept for biodiesel delivery and to assess nanosphere stability over the 150-day period.

Furthermore, EE is influenced by several variables, including polymer concentration, organic solvent and stabilizer selection, the dispersed-to-continuous phase ratio, the solvent removal rate, and the chemical properties and polymer interactions of the active ingredient itself.^48,49^ In the case of AA, the low observed EE (∼18.8%) is primarily attributed to its high hydrophilicity. This polarity promotes partitioning into the external aqueous phase during synthesis, a well-documented challenge for such compounds.^50,51^

Several strategies for improvement are proposed. First, increasing PCL concentrations during the double emulsion process may enhance organic phase viscosity, thereby slowing AA diffusion into the polar phase before solvent evaporation.^49,52^ Second, inducing a “salting-out” effect by adding electrolytes (e.g., NaCl) to the external water phase increases the medium’s ionic strength. This shift makes it thermodynamically unfavorable for hydrophilic AA to migrate out of the nanospheres.^53,54^ Finally, using more volatile organic solvents could accelerate PCL shell hardening, narrowing the temporal window for AA diffusion into the aqueous phase.^53^

Figure 1 shows the Scanning Electron Microscopy (SEM) images of the NS. Accurate identification of individual nanoparticles was challenging, likely due to sample charging under the electron beam, which precluded high-resolution imaging. Nonetheless, the images reveal relatively agglomerated polymer structures, an unfavorable characteristic for system stability. Such aggregation was expected, as the SEM samples needed to be solvent-free, and the absence of a liquid solvent reduces particle dispersion.

**Figure 1:**
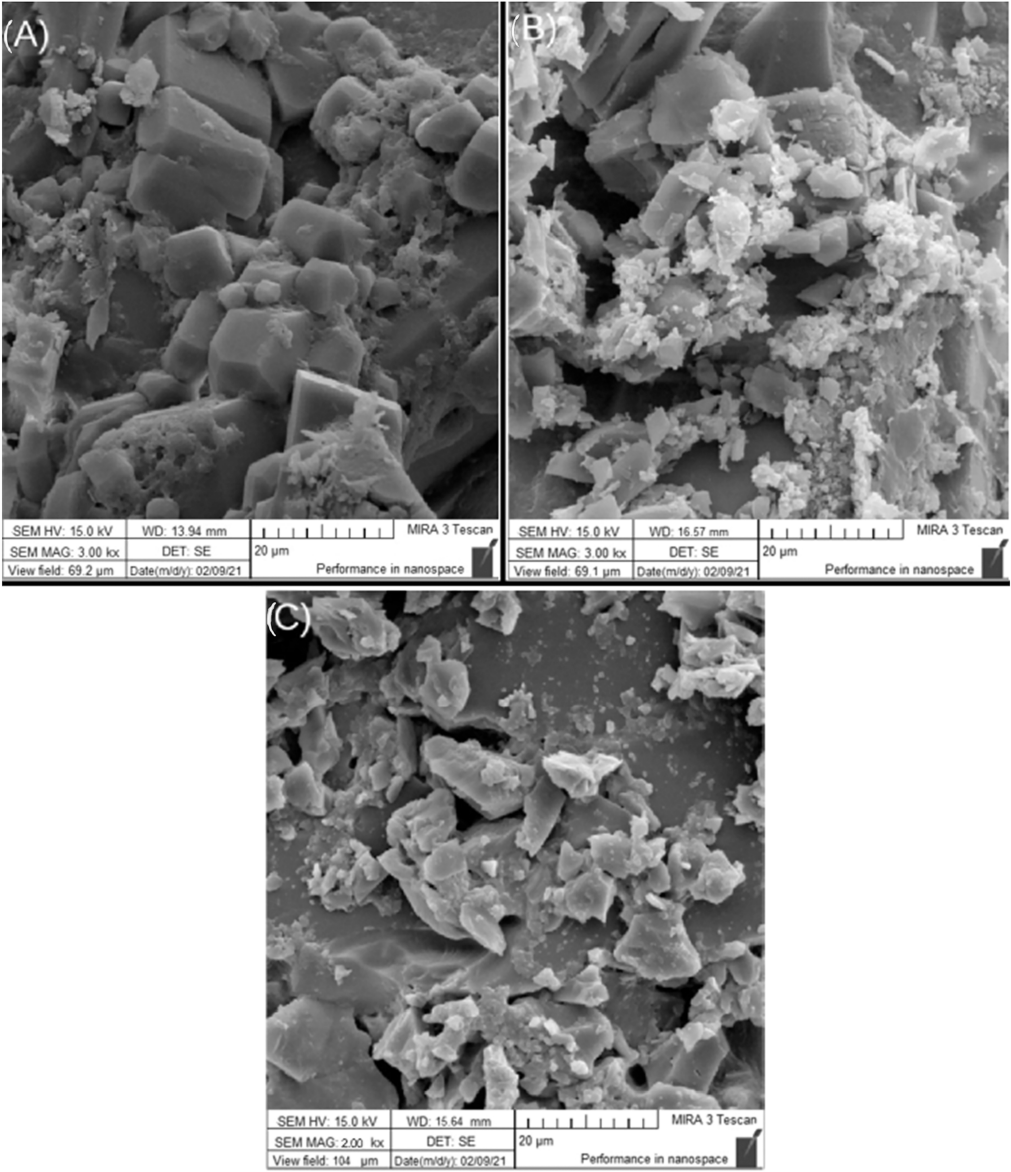
SEM images of NS containing: (A) Blank, (B) TBHQ, and (C) AA.

Furthermore, the resolution of the SEM imaging was insufficient to allow for a detailed analysis of the nanosphere surface morphology. Consequently, the acquisition of higher-resolution imaging (*e.g*., FE-SEM or HR-TEM) is recognized as an essential requirement for future studies to fully characterize the surface topography.

### 2.1 Oven Stability Test

Figure 2 presents the results of the physicochemical characterization and antioxidant activity for three samples: blank and TBHQ NS, and TBHQ aqueous solution.

**Figure 2:**
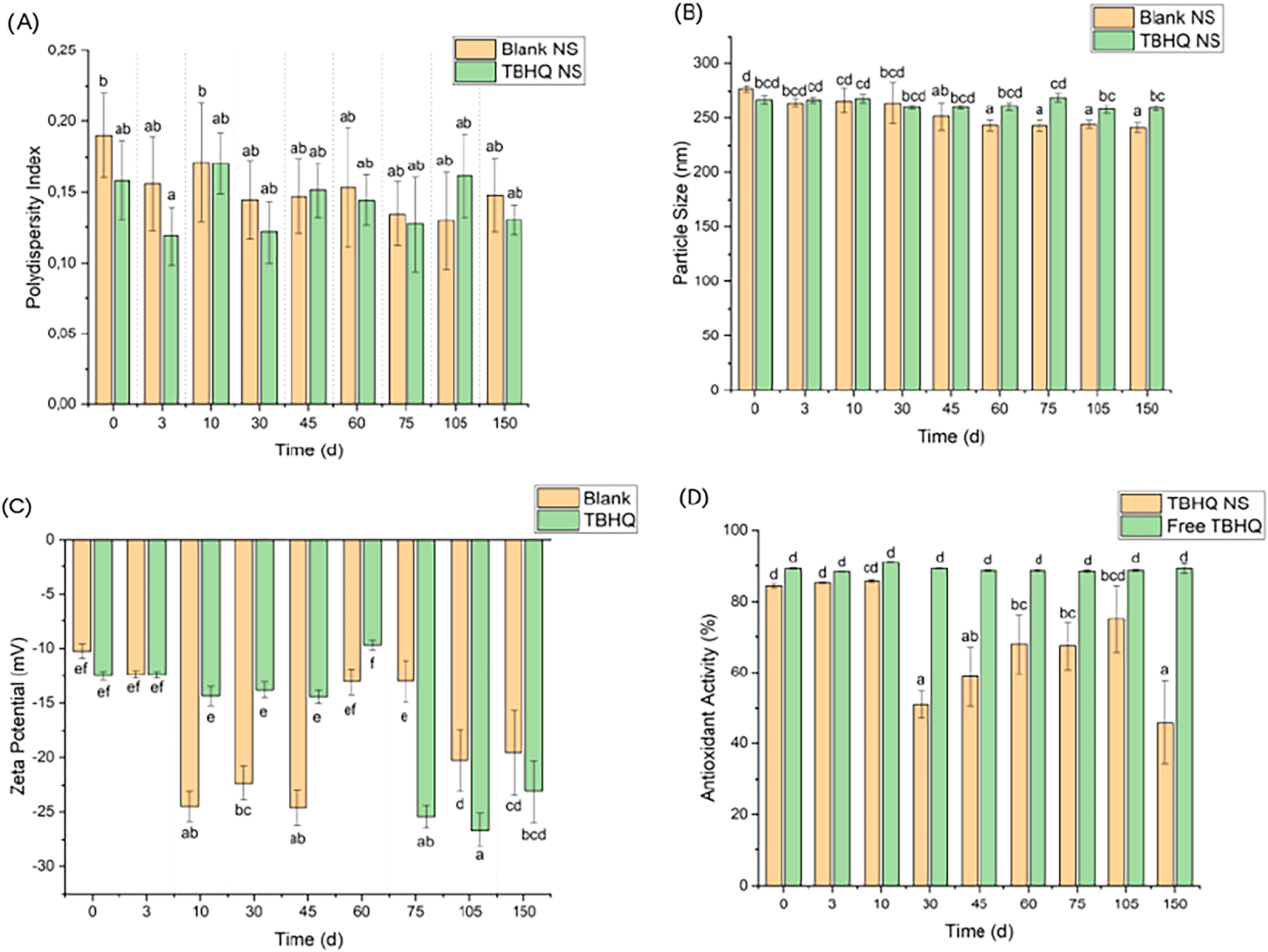
Results of: (A) PDI, (B) size, (C) zeta potential, and (D) antioxidant activity for TBHQ and blank NS, and the free TBHQ aqueous solution. Data are presented as mean values ± standard deviation (*n* = 3). Different lowercase letters at the same time interval indicate statistically significant differences between groups (one-way ANOVA, Tukey’s HSD test, *p* < 0.05).

As shown in Figure 2A, PDI results indicated that blank and TBHQ NS remained stable over 150 days, maintaining their homogeneity even under thermal stress. Similarly, particle sizes for both systems showed no significant changes, as illustrated in Figure 2B. Conversely, zeta potential measurements (Figure 2C) diverged significantly between the two suspensions. While the blank NS remained stable, the TBHQ-loaded NS exhibited notable shifts, particularly on day 60 (−9.69 ± 0.48 mV) and day 75 (−25.43 ± 1.13 mV). This shift is likely attributable to TBHQ release, where the antioxidant may have adsorbed onto the nanoparticle surface and altered its charge. This hypothesis aligns with Varan and Bilensoy,^55^ who demonstrated similar zeta potential changes when hydroxypropyl-β-cyclodextrin was present on a nanoparticle surface.

Figure S1 shows that the NS pH remained stable for most of the study. However, a drop in pH was observed on day 150. This change can possibly be caused by the formation of acidic products from polymer degradation.^56,57^ Additionally, as shown in Figure 2D, the antioxidant activity of the TBHQ aqueous solution, determined by DPPH radical reduction assays, remained above 80% throughout the 150-day thermal stress period. This result aligns with the widespread use of TBHQ as an efficient antioxidant.

It is worth noting that the antioxidant activity of the NS was evaluated using the DPPH radical scavenging assay. While the DPPH assay is typically performed in polar media, it remains a valuable standardized tool for quantifying the intrinsic radical-scavenging potential of antioxidants.^58,59^ Although the DPPH assay provides a reproducible basis for monitoring how antioxidant capacity evolves over time, it does not fully replicate the hydrophobic biodiesel matrix. Consequently, these results reflect the system’s chemical reactivity rather than providing a direct validation of performance in a nonpolar environment, and antioxidant activity analyses in nonpolar matrices are necessary to confirm the NS systems applicability to biodiesel.

In this context, the introduction of PCL nanospheres, synthesized in aqueous phases, into a nonpolar biodiesel matrix introduces a significant solvent polarity mismatch. In aqueous media, these nanospheres maintain structural integrity due to the hydrophobic effect, which stabilizes the polymer core.^60^ However, upon introduction into a nonpolar medium such as biodiesel, this stabilizing force is diminished. This environmental shift may promote polymer matrix swelling or surface reorganization, which may influence the diffusion-controlled release of encapsulated antioxidants.^61^ Furthermore, the interfacial tension between the particle surface and the biodiesel may hinder uniform dispersion and the formation of a stable colloidal suspension.^62,63^ Collectively, these interfacial and thermodynamic factors suggest a fundamental limitation for achieving effective controlled release within a biodiesel matrix.

Moreover, as shown in Figure 2D, the antioxidant activity of the TBHQ NS from day 0 to day 10 was similar to that observed for the TBHQ solution, likely due to the presence of free TBHQ in the formulation. A significant decrease in this parameter for the TBHQ-loaded NS followed after day 31, suggesting the consumption of free TBHQ rather than the release of encapsulated agents. Subsequently, the antioxidant activity gradually increased from day 45 to day 105, aligning with the sustained release profiles reported for PCL systems, extending up to one year.^64^ The antioxidant activity declined after day 105, which may stem from the degradation of the released antioxidant or its consumption by PCL degradation products. Finally, the overall antioxidant activity of the TBHQ-loaded NS remained lower than that of the free solution (Figure 4D), potentially reflecting a lower concentration of released TBHQ compared to the initial concentration of the free solution.

It is important to note that the release of the active compounds was monitored via antioxidant activity assays rather than direct mass quantification in this study. While the measured antioxidant activity is assumed to be proportional to the concentration of active TBHQ and AA in the medium, these assays provide an indirect profile of functional availability rather than a direct measurement of release kinetics. The data suggest that the antioxidant species remain active and available throughout the 150-day storage period, which is the primary requirement for biodiesel stabilization. This approach is consistent with previous studies, such as Zhou et al.,^65^ who used kinetic consumption rates and induction periods as proxies for sustained performance, and Ricci et al.^66^ who utilized TAC assays to confirm antioxidant availability. However, it must be stated that antioxidant activity assays do not directly quantify the physical mass transfer of the molecules. A comprehensive kinetic profile would require direct analytical quantification (e.g., via HPLC or UV–vis calibration in the biodiesel matrix) to definitively separate the rate of molecular release from the rate of antioxidant consumption, as the antioxidants may degrade or change forms. Consequently, the results are presented as a measure of sustained antioxidant efficacy over time.

Figure 3 shows the UV–vis absorption spectroscopy results for blank NS, TBHQ solution and TBHQ NS.

**Figure 3:**
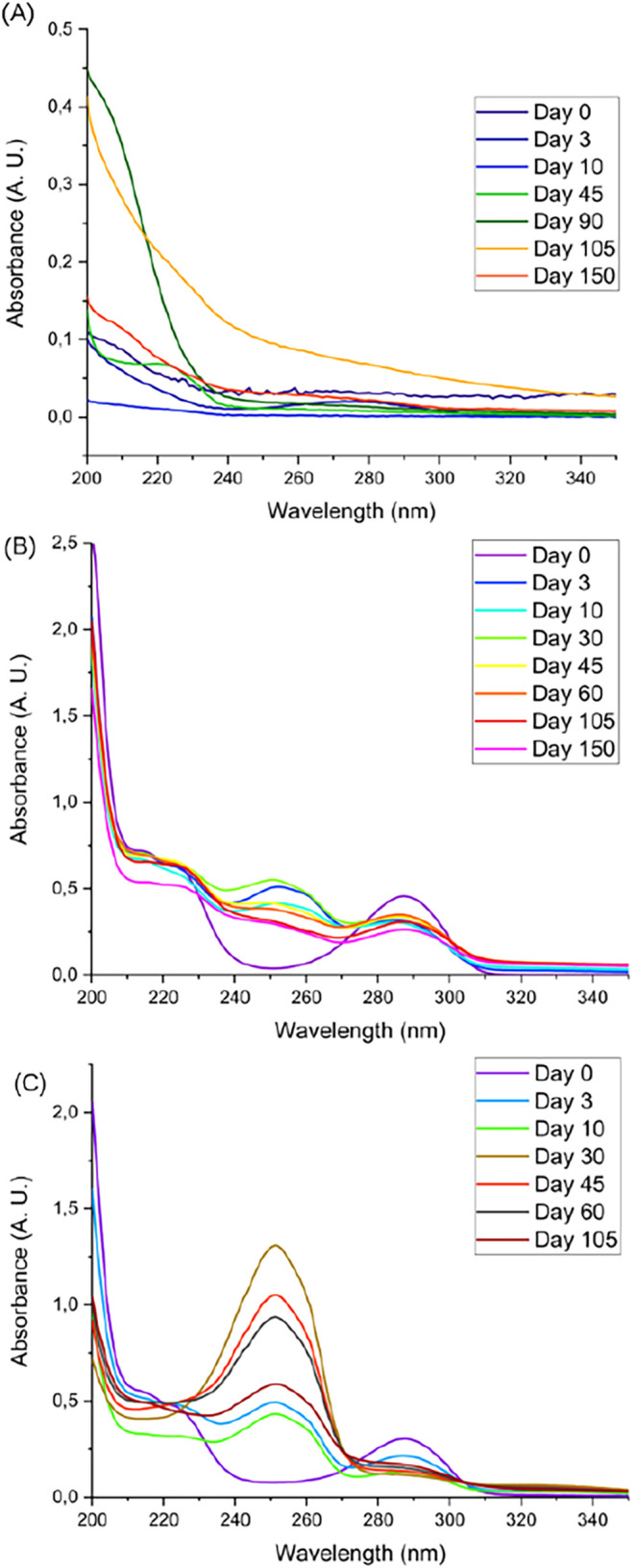
UV–vis absorption spectra for blank NS (A), Free TBHQ solution (B), and TBHQ NS (C).

A band at 287 nm is observable in the spectra of both the aqueous solution and the TBHQ-loaded NS (Figure 3B,C). This band, which shows decreasing intensity over time, is consistent with the reported maximum absorption wavelength for TBHQ (290–292 nm).^67,68^ From day 3, a new band at 250 nm appears in the spectra of both the solution and the NS suspension, with greater intensity in the TBHQ-loaded NS suspensions. The appearance of this new band indicates the presence of another compound, which may be a product of the thermal degradation of TBHQ, possibly involving multiple structures capable of absorbing in the UV–vis region, as reported by Hamama and Nawar.^69^ Consequently, the combined effects of TBHQ degradation and the gradual release of the encapsulated agent provide a plausible explanation for the lower antioxidant activity of the TBHQ NS compared to the free aqueous solution.

Furthermore, as shown in Figure 3B and 3C, the characteristic TBHQ band at 287 nm decreases over time, while the new band at 250 nm becomes gradually more prominent. Because the maximum absorption wavelength for *tert*-butyl-1,4-benzoquinone (TBBQ), the main degradation product of TBHQ, is reported at 252 nm,^67^ this spectral shift is consistent with the conversion of TBHQ into TBBQ.

Figure 4 summarizes the physicochemical properties and antioxidant activity for the AA and blank NS, as well as an AA aqueous solution.

**Figure 4:**
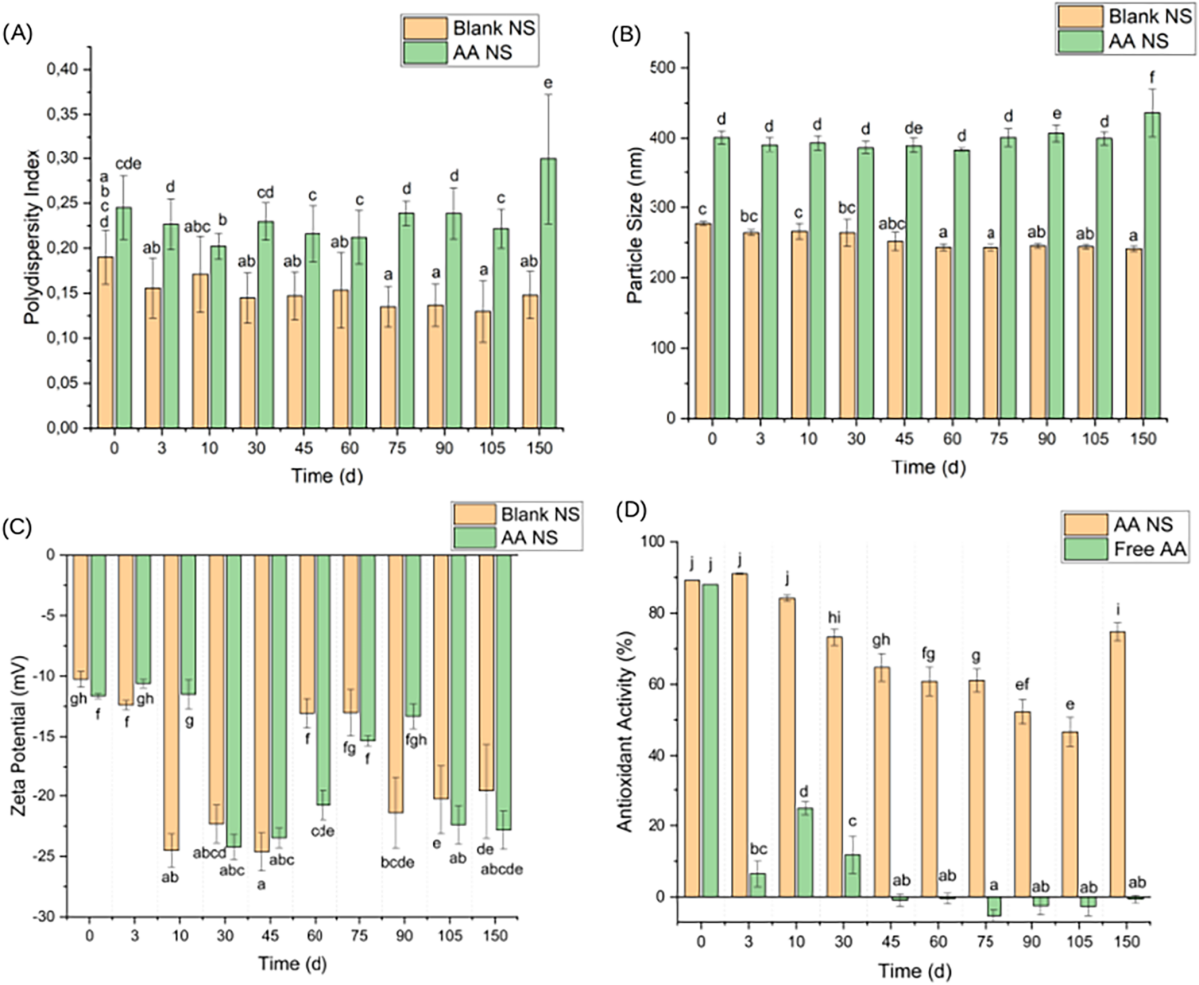
Results of: (A) PDI, (B) size, (C) zeta potential, and (D) antioxidant activity for blank and AA NS and for the AA aqueous solution. Data are presented as mean values ± standard deviation (*n* = 3). Different lowercase letters at the same time interval indicate statistically significant differences between groups (one-way ANOVA, Tukey’s HSD test, *p* < 0.05).

The AA-loaded NS effectively protected and gradually released the active compound. The NS retained more than 50% of its antioxidant activity for up to 150 days, outperforming the AA aqueous solution. Moreover, the antioxidant activity of the AA-loaded NS exceeded 90% (Figure 4D), underscoring their strong potential as stabilizing agents in biodiesel applications

Additionally, PDI and particle size results (Figure 4A,B) demonstrate long-term homogeneity over 150 days. As shown in Figure S2, pH values remained stable. This evidence corroborates the hypothesis that the NS polymer did not undergo pronounced degradation. While a pH increase would be expected in the free solution due to AA degradation, this was not observed in the nanosphere suspension, which is consistent with the protection of the active compound by the NS. Finally, the observed size and zeta potential of the AA-loaded NS align with literature values, such as the 321.8 nm diameter and −11 mV potential reported by Amin et al.^70^ for AA-loaded PCL nanoparticles.

Figure 5 illustrates the UV–vis absorption spectroscopy results for the free AA solution and AA NS. A peak at 264 nm was observed in the spectra of both the free AA solution (Figure 5A) and AA-loaded NS (Figure 5B). This band is consistent with the reported maximum absorption wavelength for AA (265 nm).^71^ Notably, this peak was no longer detectable in the free AA solution after day 1 (Figure 5B), suggesting rapid degradation of the compound. This interpretation aligns with the sudden drop in antioxidant activity observed for the free solution in Figure 4D.

**Figure 5:**
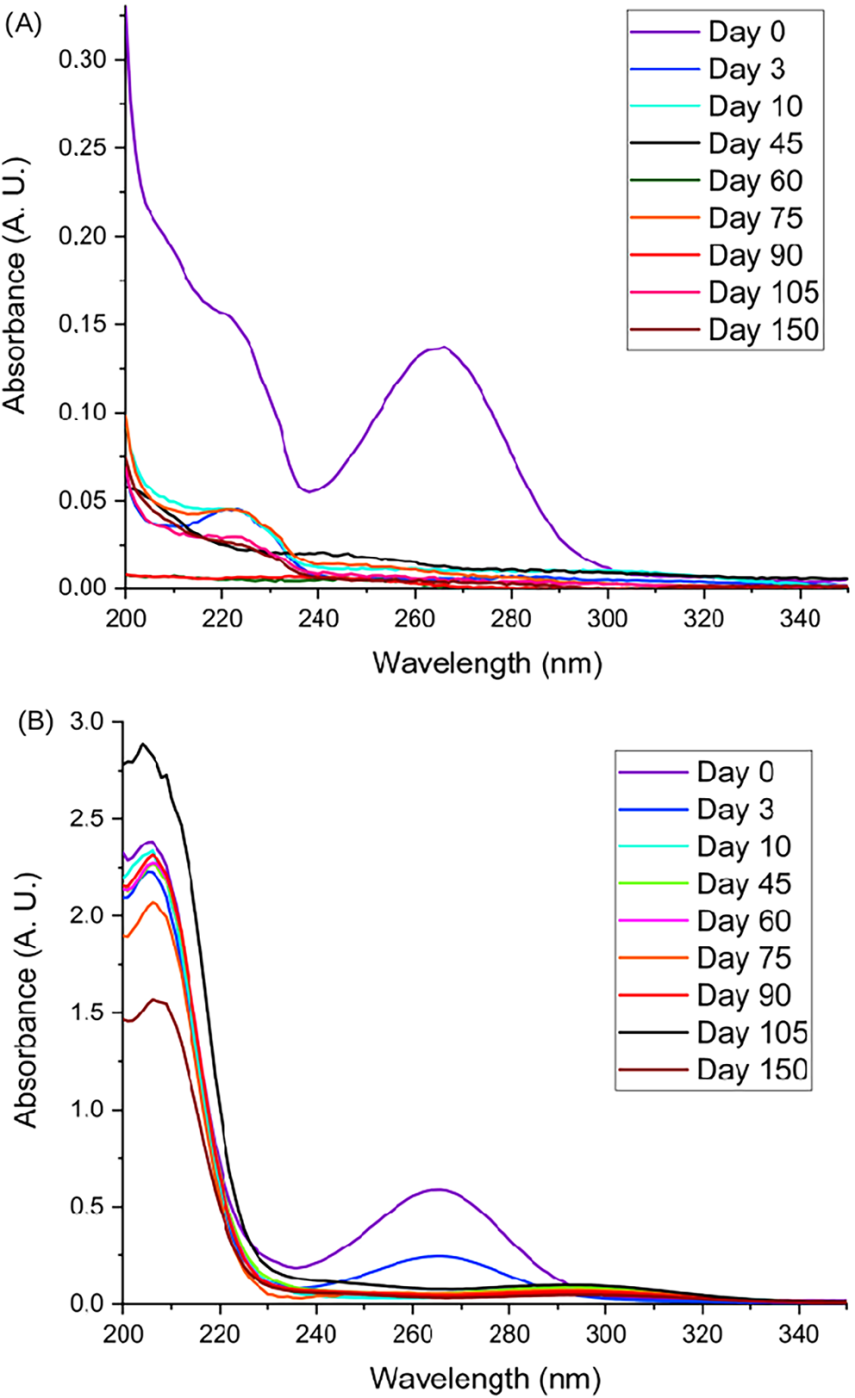
UV/vis scan of the Free AA solution (A) and AA NS (B).

In contrast, the AA-loaded NS suspension maintained the peak at 264 nm until day 3. While this specific peak subsequently disappeared, antioxidant activity persisted throughout the 150-day period, albeit at lower levels. This leads to the hypothesis that the degradation of AA was not complete. The emergence of a new peak at approximately 205 nm, near the blank NS signal (<212 nm), suggests a stable association between AA and the PCL matrix. Although AA degradation products typically absorb in the 230–300 nm region,^72^ the sustained antioxidant activity suggests alternative stabilization pathways. The intensity of the 205 nm peak remained nearly constant for most of the study, reaching its minimum on day 150. This decrease may reflect a gradual reduction of AA within the AA-PCL structure, correlating with the longitudinal decline in antioxidant activity (Figure 4D).

#### 2.1.1 Comparison Study of TBHQ and AA-Loaded NS

Figure 6 illustrates the antioxidant activity of TBHQ NS and AA NS during the stability study. Both samples maintained high antioxidant activity (above 80%) during the initial 10 days. Following this period, a reduction was observed in both systems at day 30. While the TBHQ NS exhibited a moderate recovery between days 45 and 105 before declining to its lowest recorded value by day 150, the AA NS maintained significantly higher antioxidant activity (*p* < 0.05) than the TBHQ NS at the conclusion of the study.

**Figure 6:**
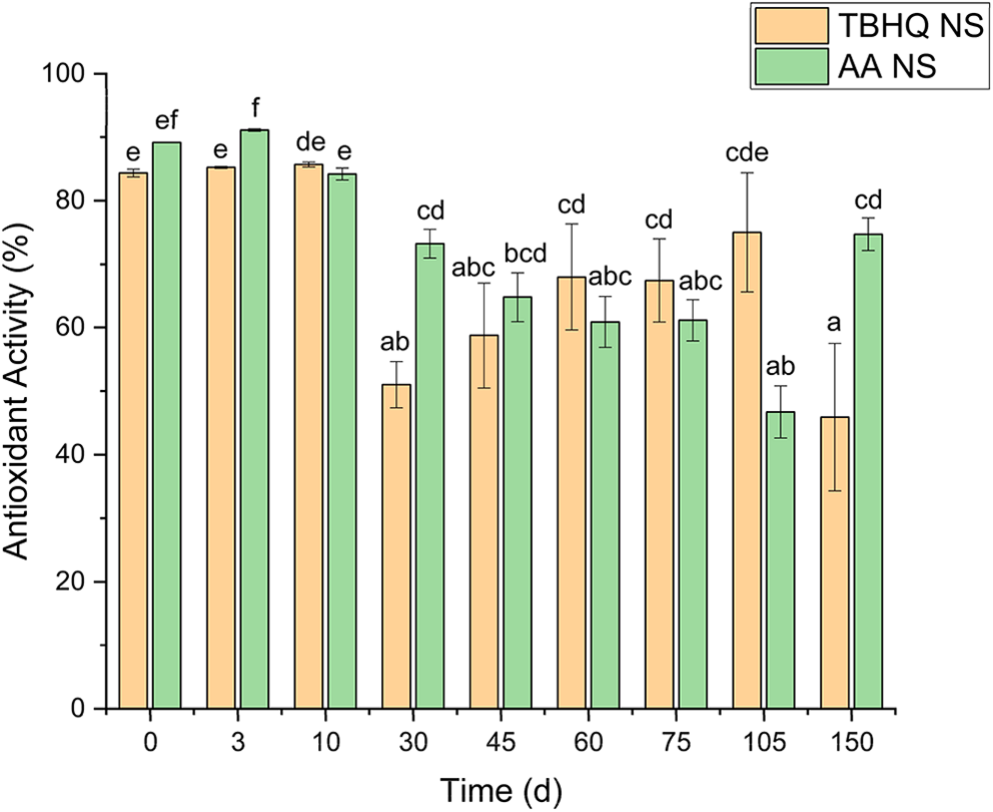
Antioxidant activity of TBHQ NS and AA NS. Data are presented as mean values ± standard deviation (*n* = 3). Different lowercase letters at the same time interval indicate statistically significant differences between groups (one-way ANOVA, Tukey’s HSD test, *p* < 0.05).

These findings align with previous reports. For instance, Franco et al.^73^ synthesized chitosan/tripolyphosphate nanoparticles loaded with *Vaccinium myrtillus* (blueberry) extract. The authors observed an initial DPPH inhibition of 34.62% for the loaded nanoparticles, which was significantly lower than the 78.99% recorded for the free extract. However, after 90 days of storage at 45 °C, both systems exhibited comparable antioxidant activity.^73^

Similar results were reported by Lozada-Ramírez et al.,^74^ who utilized ZnO nanoparticles to stabilize anthocyanins extracted from coffee husks via surface conjugation. Following 12 weeks of storage at room temperature, the conjugated systems demonstrated remarkable stability, retaining 95% of their total phenolic content and 86% of their initial antioxidant activity.

Furthermore, da Rosa et al.^75^ encapsulated *Origanum vulgare* L. essential oil within zein nanocapsules. After 90 days of storage at 20 °C, the nanocapsules retained approximately 25% of their initial antioxidant activity. In a related study, Savadkouhi et al.^76^ developed nanocapsules using *Lepidium perfoliatum* seed gum and *Orchis mascula* to encapsulate *Hyssopus officinalis* L. extract. These nanocarriers were applied on soybean oil and, after 40 days of storage at 60 °C, the samples treated with nanocapsules exhibited a thiobarbituric acid (TBA) value of 1.8 mg MDA/kg. This value was significantly lower than those recorded for both the free extract and control samples, thereby confirming the superior antioxidant efficacy of the encapsulated system.^76^

Similarly, Jafari et al.^77^ encapsulated *Rosmarinus officinalis* L. leaf extracts within *Ocimum basilicum* seed gum nanocapsules and evaluated their protective effect on sunflower oil. Following a 24-day storage period at 60 °C, the samples with the nanoencapsulated extract had a TBA value of 0.06 μg/mL, lower than samples with free extract.^77^

In a separate study, de Farias et al.^78^ nanoencapsulated carotenoids from *Nannochloropsis oculata* in chitosan-based nanoemulsions. Using the ABTS radical scavenging method over a 30-day storage period at 25 °C, the authors found that the formulation prepared with 1.00% chitosan exhibited remarkable stability, retaining 90% of its initial ABTS inhibitory capacity.^78^

Additionally, Taghadosi et al.^79^ used Persian gum-based nanocapsules to encapsulate curcumin and evaluated their effect in a yogurt matrix. After 21 days of refrigerated storage, the DPPH antioxidant activity of samples containing 3% (w/w) of curcumin nanocapsules decreased only marginally, from 69.95% to 68.65%, indicating high stability and protective efficacy.^79^

Finally, Hosseini et al.^80^ encapsulated chia seed extract within mixed lecithin and basil seed gum nanocapsules and evaluated their effect on ricotta cheese. After 15 days of storage at 4 °C, the DPPH antioxidant activity of the samples containing 3.00% (w/w) nanocapsules decreased from an initial 94.81% to 77.99%.^80^

### 2.2 Oxidation Stability Studies

To assess the *in situ* antioxidant effect of the NS, two different approaches were employed to incorporate them into biodiesel. In the first method, the NS suspension was introduced during the final biodiesel washing step. In the second, the lyophilized NS powder was directly dispersed into the biodiesel after washing under mechanical stirring. As shown in Table 2, the presence of the NS accelerated biodiesel oxidation. This is evident as the induction time (IT) was lower than that of the biodiesel without any added antioxidants. This outcome contrasts sharply to the *in vitro* radical scavenging tests, which showed satisfactory antioxidant activity.

**Table 2:** Induction Time for Biodiesel Samples with and without the Addition of NS-Containing Antioxidants (*n* = 3)

Sample	Lyophilized (h)	Nonlyophilized (h)
AA NS	3.56 ± 0.01	*4.71 ± 0.13*
TBHQ NS	3.48 ± 0.1	*4.77 ± 0.04*
Blank NS	3.25 ± 0.41	-
Pure Biodiesel	5.87 ± 0.14	

Studies that analyzed the effects of nanoparticles on the oxidative stability of biodiesel remain exceptionally scarce. Among these, Torkzaban et al.^81^ synthesized carbon quantum dots from pomegranate peels and evaluated their effectiveness as additives for waste cooking oil biodiesel. Their results indicated an IT of 92.21 h, alongside improved engine performance and reduced emissions.^81^ Similarly, Kapile et al.^82^ investigated the influence of silica nanoparticles extracted from rice husk on *Adansonia digitata* biodiesel. They reported an increase in IT from 3.8 to 10.3 h for biodiesel samples containing 800 ppm of nanoparticles.^82^ Notably, in both cases, the nanoparticles themselves exhibited inherent antioxidant activity.

The observed behavior in the current study suggests that the antioxidant was not released as expected, a result that may be attributed to the polarity of the medium. The previous *in vitro* tests were conducted in an aqueous solution, whereas biodiesel is a nonpolar, organic medium. The significant difference in polarity between these media likely inhibited the dissolution and dispersion of the NS, thereby preventing the release of the active compounds. Another reason for the lack of an increase in IT may be related to the method of adding the NS to the biodiesel. The incorporation was performed without employing methods capable of ensuring complete homogenization, such as heating and ultrasound, and this incomplete homogenization could have prevented the effective release and activity of the antioxidant. As mentioned before, given the aqueous-based synthesis, achieving optimal colloidal stability in a hydrophobic medium is inherently challenging.

One hypothesis for the observed reduction of the IT compared to pure biodiesel is relates to the NS morphology. As the NS usually deviate from perfect sphericity, they may possess surface imperfections at the nanometric scale, reminiscent of those in zeolites, which may have served as active sites promoting radical oxidation reactions. Moreover, the role of similar active sites in zeolites as heterogeneous catalysts for biodiesel production,^83,84^ and as catalysts for other reactions, such as the catalytic pyrolysis of kraft lingin,^85^ is well-documented in the literature.

However, given the current lack of NS surface analyses, such as Brunauer–Emmett–Teller (BET) analysis, Fourier-transform infrared spectroscopy (FTIR), X-ray photoelectron spectroscopy (XPS), and transmission electron microscopy (TEM) lattice imaging, this explanation remains a hypothesis that requires further experimental validation.

Moreover, the incomplete homogenization may have been responsible for the IT reduction, either in part or completely. It is acknowledged that potential particle aggregation or nonuniform distribution could introduce experimental variability. Nevertheless, the reproducibility of the observed pro-oxidant behavior across all trials suggests that the phenomenon is also explainable by the hypothesis of physicochemical interaction at the nanosphere surface, rather than solely by aggregation-related effects.

Additionally, it is acknowledged that the present study relied solely on IT as a metric for biodiesel oxidation stability. While the Rancimat method is the industry standard for determining the end of the induction period, this approach provides limited insight into the intermediate stages of the oxidation process. The simultaneous quantification of peroxide values (to monitor primary oxidation products), acid values (to track the formation of secondary carboxylic acids), and GC-MS analysis (to identify specific volatile degradation species) would have provided a more granular understanding of the oxidation kinetics. The absence of these complementary analytical techniques is recognized as a limitation, and their inclusion is proposed as a necessary focus for future research.

Another noteworthy effect of the NS over biodiesel was a change in the oxidation kinetics. Samples with NS followed second-order kinetics, while pure biodiesel samples followed first-order kinetics, as shown in Table 3. This shift in reaction order may result from the influence of NS on the IT. Upon adding the NS, the biodiesel oxidation reaction possibly became influenced by multiple components and factors, thus causing it to become a second-order reaction.^86^

**Table 3:** Linear Regression Analysis of the Integrated Rate Laws for Zero-, First-, and Second-Order Reaction Kinetics

Sample	Reaction order	*R* ^2^	Intercept	Slope
Blank NS + biodiesel	0	0.82709	0.03082	–1.09256 × 10^–6^
	1st	0.94954	–2.81532	–1.00258 × 10^–4^
	*2nd*	*0.99799*	–*77.75576*	*0.01061*
AA NS + biodiesel	0	0.94056	0.02952	–1.11779 × 10^–6^
	1st	0.98824	–2.38262	–1.29699 × 10^–4^
	*2nd*	*0.99857*	–*170.81933*	*0.01603*
TBHQ NS + biodiesel	0	0.88859	0.03548	–1.33836 × 10^–6^
	1st	0.97539	–2.23375	–1.29761 × 10^–4^
	*2nd*	*0.99853*	–*148.06671*	*0.01411*
Pure biodiesel (control)	0	0.99146	0.02583	–3.86223 × 10^–7^
	*1st*	*0.99936*	–*3.43602*	–*2.75633 × 10^–5^*
	2nd	0.99805	11.99919	0.00199

### 2.3 Future Research Directions

This study highlights several potential avenues for future research. Alternative encapsulation methods for AA should be explored to improve its entrapment efficiency. Furthermore, supplementing the antioxidant activity assays during the stability test with direct chemical quantification, such as LC-MS, GC-MS, or HPLC, would be a significant improvement, enabling the modeling of release kinetics and the elucidation of precise reaction pathways.

Additionally, future stability tests could be conducted in an apolar medium that better simulates the chemical environment of biodiesel to refine the assessment of the delivery system, with tests such as Oxygen Radical Absorbance Capacity (ORAC), ABTS, and lipid peroxidation.

Moreover, future work should explore surfactant-assisted dispersion protocols and other homogenization methods, such as high-shear methods or sonication, to properly homogenize the NS suspensions into the biodiesel matrix, thus eliminating experimental variability. Future investigations should also involve comparative efficacy studies in biodiesel against free antioxidants (*e.g*., TBHQ, AA) to decouple the effects of the polymer matrix from the antioxidant payload.

Furthermore, future investigations into biodiesel oxidation stability should supplement the Rancimat IT with simultaneous quantifications of peroxide values and acid values, alongside GC-MS analyses. Such a multiparameter approach is essential to provide deeper insights into the specific stages of the oxidation process, monitoring the formation of primary and secondary degradation products, and to fully elucidate the mechanistic influence of nanosphere-encapsulated antioxidants on fuel stability.

Finally, a thorough investigation of the NS surface chemistry, employing techniques such as BET, FTIR, XPS, and TEM lattice imaging analyses, is required to definitively characterize the surface defect pro-oxidant catalysis hypothesis and to determine whether molecular interactions or changes in crystallinity occur upon encapsulation.

Regarding the industrial scalability of this nanosphere-based antioxidant delivery system, several technical challenges must be addressed for large-scale commercialization. First, the current laboratory-scale synthesis, while effective for proof-of-concept, involves organic solvent phases that require sophisticated solvent recovery or sustainable aqueous-based synthesis routes to meet industrial environmental standards. Second, the EE, particularly for hydrophilic compounds such as AA, represents a potential bottleneck. As such, optimizing EE is essential to improve the economic viability of the final product. Furthermore, while the current study demonstrates physical and functional stability over 150 days under controlled conditions, industrial implementation will require rigorous testing of nanosphere integrity under high-shear conditions typical of industrial pumping and storage processes. Moreover, a concentration optimization study to analyze the effect of the concentration of NS formulations on biodiesel is necessary to understand the necessary dosage, a critical factor for industrial scalability. However, scalability challenges are well-documented in the development of nanocarriers for other sectors, most notably drug delivery, where these limitations remain the subject of intensive research.^87^

## 3 Conclusions

In conclusion, TBHQ and AA were successfully encapsulated within PCL nanospheres, achieving high physicochemical stability and sustained antioxidant activity retention over an extensive 150-day period at 30 °C. Specifically, the TBHQ NS maintained a DPPH scavenging antioxidant activity at over 80%, while the AA NS maintained greater than 50% antioxidant activity in these conditions, and both NS formulations consistently maintained a PDI value under 0.3. Despite this robust *in vitro* performance, their application in biodiesel reduced induction time compared to the pure biofuel control. The induction time decreased from 5.87 ± 0.14 h for pure biodiesel to 4.77 ± 0.04 h for TBHQ NS and 4.71 ± 0.13 for the AA NS. Furthermore, the biodiesel oxidation kinetics shifted from a first-order to a second-order model upon the incorporation of NS. This unexpected pro-oxidant effect is hypothesized to result from nanometric surface imperfections on the NS acting as catalytic sites, exacerbated by suboptimal dispersion in the nonpolar medium. The combination of a possible catalytic surface activity and suboptimal distribution may have accelerated the oxidative process rather than inhibiting it. Therefore, a valid future research avenue is the confirmation of this hypothesis with further analyses and, if confirmed, the precise engineering of nanoparticle morphology and surface topography, as optimizing these structural parameters may prevent unintended catalytic interactions and ensure the successful deployment of polymeric carriers in hydrophobic fuel matrices. Finally, as the *in vitro* antioxidant activity assays were conducted in polar media, further validation in apolar systems is necessary to confirm the applicability of the nanosphere system to biodiesel.

## 4 Materials and Methods

### 4.1 Reagents

The following reagents were used in this research: >99.5% Dichloromethane, from Reagen Reatec (Colombo, Paraná, Brazil); 97% 2,2-diphenyl-1-picryl-hydrazyl (DPPH), from Tokyo Chemical Industry (Tokyo, Japan); >99.9% dimethylsulfoxide (DMSO), from Synth (Diadema, São Paulo, Brazil); 99.8% methanol, from Êxodo Científica (Sumaré, São Paulo, Brazil); >85% potassium hydroxide (KOH), from Alphatec (Porto Alegre, Rio Grande do Sul, Brazil); 36.5–38.0% chlorine acid (HCl), from Dinâmica Química Contemporânea (Indaiatuba, São Paulo, Brazil); 99.8% NaCl, from Neon (Suzano, São Paulo, Brazil). Furthermore, the antioxidants utilized were >99.0% ascorbic acid, from Synth (Diadema, São Paulo, Brazil), and 97% TBHQ, from Sigma-Aldrich (Saint Louis, USA). Finally, the used polymers were average *M*_w_ 80.0 PCL and *M*_w_ 89.0–98.0 Poly­(vinyl alcohol) (PVA), both from Sigma-Aldrich (Saint Louis, USA).

### 4.2 Preparation of AA Polymeric NS

NS were produced using a double emulsion solvent evaporation technique.^88^ As presented in Figure 7, the process involved two aqueous phases and one organic phase. The organic phase was prepared by dissolving 50 mg of PCL in 1.8 mL of dichloromethane. This solution was then poured into a solution of 10 mg of AA dissolved in 0.2 mL of DMSO. The first emulsion was created by dripping the organic phase into the first aqueous phase, which consisted of 4 mL of a 0.2% w/w PVA solution, under sonication. To form the second emulsion, the first emulsion was subsequently poured into the second aqueous phase, consisting of 4 mL of a 1% w/w PVA solution, under the same conditions. Finally, the dichloromethane was removed by rotary evaporation at 37 °C for 20 min.

**Figure 7:**
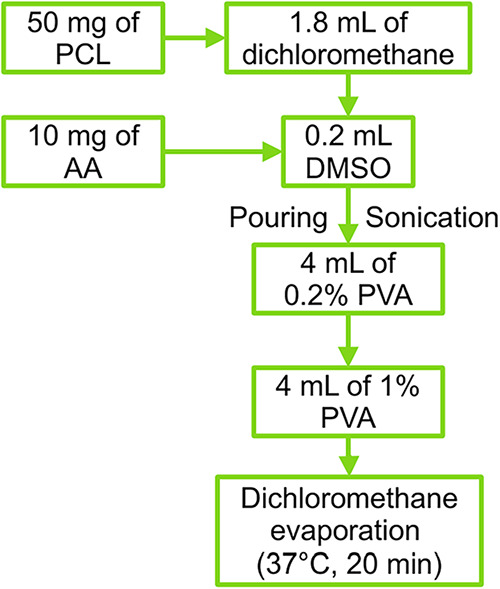
Flowchart depicting the polymeric NS preparation process.

Ten formulations of AA NS were prepared and diluted with distilled water to a final volume of 100 mL for the stability study. The solution was divided into three 30 mL aliquots, which were used for the oven stability test.

### 4.3 Preparation of TBHQ Polymeric NS

NS were prepared using the double emulsion technique,^88^ similarly to the preparation of the AA NS. Five formulations of TBHQ nanospheres were prepared. In two of these, the amount of all reagents was multiplied by three. The final volume was adjusted to 100 mL with distilled water.

### 4.4 Preparation of Active Ingredient-Free Polymeric NS

Blank NS, which are NS without an active ingredient, were prepared using a modified version of the same emulsification and solvent evaporation protocol. The sole modification was that the organic phase for the blank NS contained only PCL dissolved in 2 mL of dichloromethane.

### 4.5 Determination of the EE of the TBHQ Nanospheres

The EE was determined indirectly by measuring the unencapsulated active substance in the supernatant. Aliquots (2 mL) of each NS suspension were centrifuged at 38,000 g for 50 min, and the supernatants were analyzed.^14^

The active substances were quantified with a Thermo Scientific Evolution 201 spectrophotometer (Waltham, USA) at a wavelength of 287 nm, using a calibration curve from aqueous solutions of TBHQ at concentrations of 2, 4, 6, 10, and 20 mg·L^–1^. The curve showed a strong linear relationship, with an *R* ^2^ value of 0.9991 and the equation *y* = 0.0199*x* + 0.0053. Finally, the EE was calculated according to eq 1.

1 EE(%)=[(TAI−SAI)/TAI]×100

In which TAI represents the “total active ingredient” and SAI represents the “supernatant active ingredient”.

### 4.6 Determination of the EE of the AA-Loaded NS

The EE of the AA-loaded NS suspension was determined indirectly, using the same method and conditions used for the TBHQ NS.^14^ The unencapsulated AA was quantified by a Thermo Scientific Evolution 201 spectrophotometer (Waltham, USA) at a wavelength of 264 nm. Furthermore, a calibration curve was constructed from aqueous solutions of AA at concentrations of 1, 2, 5, 7, and 10 mg·L^–1^, which yielded a strong linear fit with a 0.9997 *R* ^2^ and the equation *y* = 0.0766*x* + 0.0255. Finally, the percentage of AA incorporated into the NS was calculated according to eq 1.

### 4.7 SEM Analysis

Samples of Blank NS, TBHQ NS, and AA NS were subjected to SEM analysis using a Tescan MIRA 3 scanning electron microscope (Brno, Czech Republic) operating at an accelerating voltage of 15.0 kV. Since the polymer nanospheres are nonconductive, the samples were sputter-coated with a thin layer of gold using a Quorum Q150R ES sputter coater (Laughton, UK).

### 4.8 Oven Stability Test

#### 4.8.1 Sample Preparation for the Stability Test

To prepare the aqueous TBHQ-loaded NS suspensions, the initial 100 mL suspension (after determining its EE) was adjusted to a concentration of 500 ppm by adding 17.74 mL of distilled water. This resulting suspension was divided into three 35 mL samples, which were then stored in the oven for the study. Similarly, an AA-loaded NS suspension was prepared at a concentration of 180 ppm and divided into three 30 mL samples.

Additionally, three formulations of blank NS, without an active substance, were prepared and then adjusted to a final volume of 100 mL with distilled water in a volumetric flask. This suspension was subsequently divided into three 30 mL samples.

Furthermore, control solutions of the unencapsulated active substances, AA, and TBHQ were also prepared at the same concentrations as the NS suspensions. More specifically, a 100 mL solution of 500 ppm TBHQ was prepared and subsequently divided into three 30 mL aliquots. For AA, a 100 mL aqueous solution at 180 ppm was prepared and similarly divided into three 30 mL aliquots.

#### 4.8.2 Stability Study in an Oven at 30 °C

For the stability study, triplicate samples of the NS suspensions were stored in a SOLAB SL-100 oven (Piracicaba, São Paulo, Brazil) at 30 °C for 150 days. During this period, size, PDI, zeta potential, pH and antioxidant activity of the samples were analyzed. Samples of TBHQ NS were collected on days 0, 3, 10, 30, 45, 60, 75, 105, and 150. Meanwhile, for the AA and blank NS, samples were collected on days 0, 3, 10, 30, 45, 60, 75, 90, 105, and 150.

For comparison, the study was repeated with aqueous solutions of the pure antioxidant substances, which were analyzed only for their antioxidant activity and pH.

### 4.9 Physicochemical Characterization of NS

#### 4.9.1 PDI, Size, Zeta Potential, and pH

The PDI, average particle diameter, and zeta potential were determined in triplicate using a Malvern Nano-ZS90 Zetasizer (Malvern, UK). Dynamic light scattering was used to measure PDI and particle diameter, while electrophoretic light scattering was employed for the zeta potential analysis. Samples were prepared by diluting them with distilled water at a 1:100 (v/v) ratio. All analyzes were performed in triplicate.

Furthermore, a Tecnopon MPA 210 pH meter (Piracicaba, São Paulo, Brazil) was used to measure the pH of the aqueous suspensions. All analyzes were performed in triplicate.

### 4.10 Antioxidant Activity

The antioxidant activity of the aqueous solutions and NS suspensions was evaluated using the DPPH radical reduction method,^89^ in which DPPH is reduced to 2,2-diphenyl-1-picrylhydrazine (DPPH-H) through reactions with antioxidant compounds. This reaction results in a color change from violet to yellow and a decrease in absorbance at 515 nm.^90^ Prior to the assay, the NS suspensions were centrifuged at 38,000 g for 50 min, and the supernatants were collected. The absorbance of pure DPPH (control), NS supernatant samples, and blanks was measured at 515 nm in a Thermo Scientific Evolution 201 spectrophotometer. The percentage of antioxidant activity was then calculated using eq 2.

2 $$\mathrm{Antioxidant activity}\;\left(\%\right)=\left[\left(A_{\mathrm{control}}-A_{\mathrm{sample}}-A_{\mathrm{blank}}\right)/A_{\mathrm{control}}\right]\times 100\%$$

In which *A*_control_ represents the DPPH control solution absorbance, *A*_sample_ represents the DPPH’s absorbance with the respective sample, and *A*_blank_ represents the blank absorbance.

### 4.11 Statistical Analysis

Statistical significance for the stability results was determined using one-way analysis of variance (ANOVA) followed by Tukey’s Honest Significant Difference (HSD) posthoc test. All analyses were carried out with the Origin Software. To ensure the independence of the experimental groups across different data sets, categorical codes were assigned to each sample type at every time interval. Depending on the specific comparison, this allowed for the simultaneous evaluation of mean values between the free antioxidant solutions, Blank NS, AA NS, and TBHQ NS throughout the 150-day observation period. In all cases, differences were considered statistically significant at *p* < 0.05.^91^

### 4.12 Biodiesel Production

Biodiesel was produced via the transesterification of commercially obtained soybean oil using methanol and a KOH catalyst, as described by Gallina et al.^92^ While the produced biodiesel was not characterized, soybean based biodiesel usual fatty acid methyl ester (FAME) composition is well-known, and the usual composition is presented in Table 4.

**Table 4:** Soybean-Based Biodiesel’s Usual FAME Composition

Fatty acid	Composition (%)^a^
Linoleic acid (C18:2)	42.0–55.7
Oleic acid (C18:1)	19.0–28.1
Palmitic acid (C16:0)	9.9–12.1
Linolenic acid (C18:3)	10.2–13.3

a Data obtained from Corseuil et al.^93^

### 4.13 Preparation of TBHQ and AA NS and Their Addition to Biodiesel

Two formulations of each NS type were prepared for addition to biodiesel. The NS were incorporated using two different methods, as depicted in Figure 8. In the first method, biodiesel was washed with the NS suspension, replacing the standard aqueous washing step. In the second method, lyophilized NS, obtained using a Terroni Enterprise model II lyophilizer (São Carlos, São Paulo, Brazil), were directly added to the biodiesel. These procedures were conducted to evaluate the effects of the two addition methods on the oxidation stability of the biodiesel.

**Figure 8:**
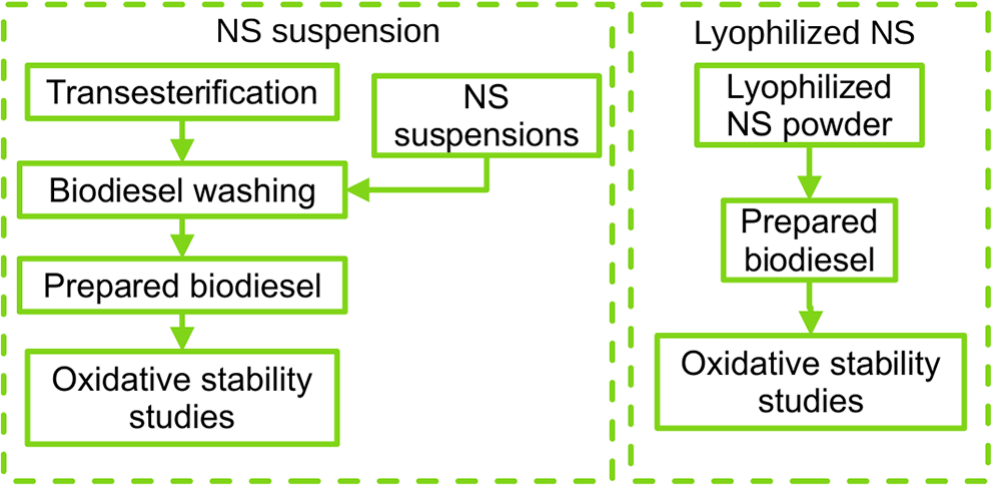
Flowchart depicting the methods for NS addition into biodiesel.

### 4.14 Evaluation of Biodiesel Induction Time and Oxidation Kinetics Study

To assess the oxidation stability of the biodiesel, its induction time was measured at 110 °C and with an air insufflation rate of 10 L/h, using a Metrohm 873 Rancimat equipment (Herisau, Switzerland), as described in the EN 14112:2016 standard.^94^ Furthermore, this measurement was performed with and without the addition of antioxidant-bearing NS.

Furthermore, the oxidation kinetics of the biodiesel samples were investigated to determine the reaction order in both the presence and absence of antioxidant-loaded nanospheres. Oxidative stability tests were performed via the Rancimat method at 110 °C. Kinetic parameters were evaluated using integrated rate laws modified according to the methodology described by Amaral et al.^13^ This approach utilizes electrical conductivity (Λ) as a proxy for the extent of biodiesel degradation, as Λ increases proportionally with the formation of volatile carboxylic acids.

The experimental data, including only data points after each respective IT, were fitted to zero-order (eq 3), first-order (eq 4), and second-order (eq 5) integrated rate equations, in which Λ represents conductivity, Λ_0_ represents initial conductivity, *k* represents the rate constant, and *t* represents time. Linear regression analysis was performed using Origin software, and the reaction order was determined based on the highest coefficient of determination (*R* ^2^). Finally, all regression analyses were performed on biodiesel samples incorporated with lyophilized NS.

3 $$\left(\frac{1}{\Lambda }\right)=\left(\frac{1}{\Lambda_{0}}\right)-\mathrm{kt}$$

4 $$\ln\left(1/\Lambda \right)=\left(\ln\left(1/\Lambda_{0}\right)\right)-\mathrm{kt}$$

5 $$\Lambda =\Lambda_{0}+\mathrm{kt}$$

## Supplementary Material



## Notes

Observed pH of free TBHQ and AA solutions, TBHQ NS, AA NS, and Blank NS over the 150-day stability study (PDF)

## §

Department of Chemistry, Autonomous University of Barcelona (UAB), Placa Civica, 08193 Bellaterra, Barcelona, Spain
