# Supplementary material for: Synthesis, In Vitro Antioxidant Activity, and Physicochemical Stability of Antioxidant-Containing Nanospheres and Their Effects on Biodiesel Oxidation Stability
Source: ACS Omega. 2026 May 19;11(21):31231–44. doi: 10.1021/acsomega.6c00993 (PMC13234901; doi:10.1021/acsomega.6c00993)
Supplement: Supplementary file 1 [file ao6c00993_si_001.pdf]

SYNTHESIS, *IN VITRO* ANTIOXIDANT ACTIVITY AND PHYSICOCHEMICAL STABILITY OF ANTIOXIDANT-CONTAINING NANOSPHERES AND THEIR EFFECTS ON BIODIESEL OXIDATION STABILITY

Supporting Information

Eduarda Carolina Hagemann Lopes[1#], Karen Cristine Silva de Oliveira[1], Giovano Tochetto[2], Fernanda Oliveira Lima[1], Dalila Moter Benvegnú[1], Letiére Cabreira Soares[1], André Lazarin Gallina[2]*

[1] Federal University of Fronteira Sul (UFFS), Campus Realeza, Postal Office Box 253, Avenida Edmundo Gaievski, 1000, Rodovia BR 182 – Km 466, postal code 85770-000, Realeza, Paraná (PR), Brazil.

[2] Department of Chemistry, Midwestern Paraná State University (UNICENTRO), Alameda Élio Antonio Dalla Vecchia, 838, Center for Technological Development of Guarapuava, postal code 85040-167, Guarapuava, Paraná (PR), Brazil.

# Current address: Department of Chemistry, Autonomous University of Barcelona (UAB), Plaça Cívica, 08193, Bellaterra, Barcelona, Spain.

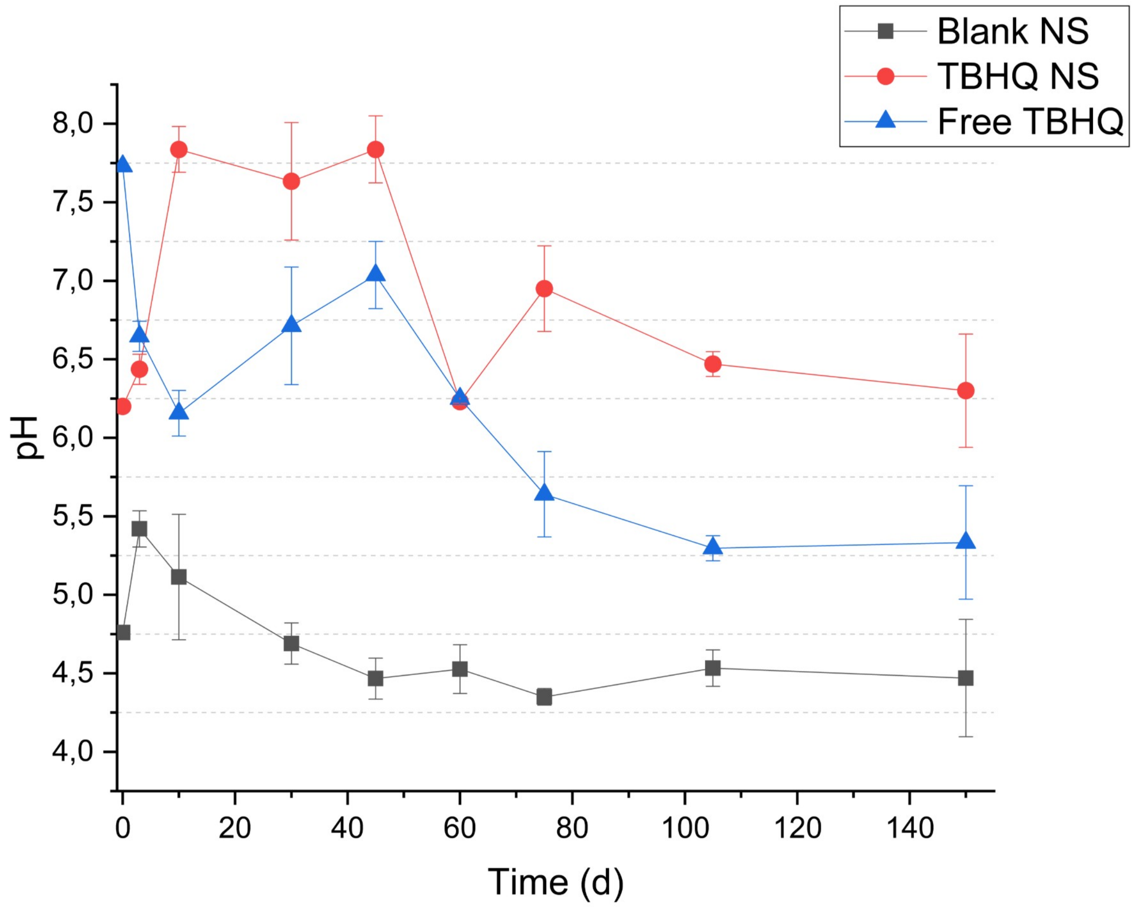

Figure S1: pH for Blank NS, TBHQ NS, and Free TBHQ solution.

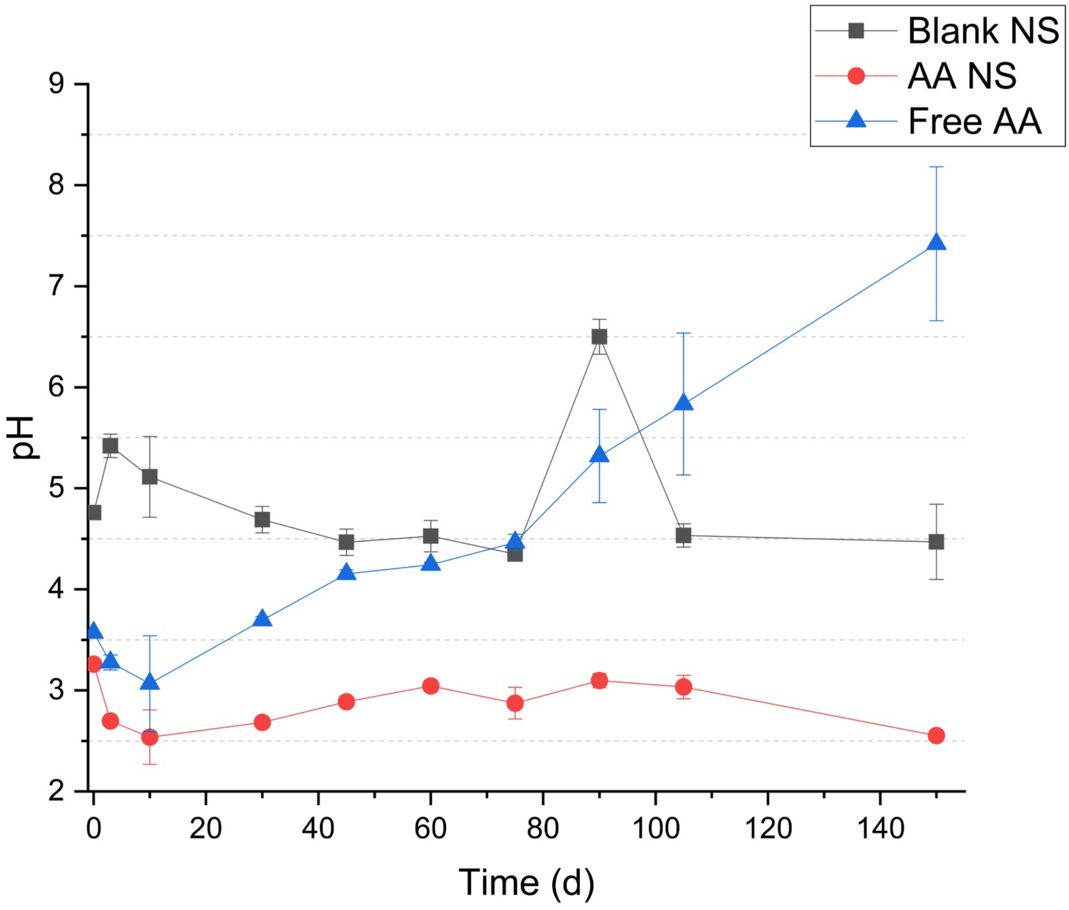

Figure S2: pH for Blank NS, AA NS, and Free AA solution.
